# n-3 PUFA Sources (Precursor/Products): A Review of Current Knowledge on Rabbit

**DOI:** 10.3390/ani9100806

**Published:** 2019-10-15

**Authors:** María Rodríguez, Pilar G. Rebollar, Simona Mattioli, Cesare Castellini

**Affiliations:** 1 Departamento de Producción Agraria, Escuela Técnica Superior de Ingeniería Agronómica Alimentaria y de Biosistemas, Universidad Politécnica de Madrid, Ciudad Universitaria s/n, 28040 Madrid, Spain; maria_rodriguez_francisco@hotmail.com; 2 Department of Agricultural, Environmental and Food Science, University of Perugia, Borgo XX Giugno, 74, 06121 Perugia, Italy; simona.mattioli@hotmail.it (S.M.); cesare.castellini@unipg.it (C.C.)

**Keywords:** PUFA sources, PUFA metabolism, supplementation, rabbit

## Abstract

**Simple Summary:**

The nutritional quality of fat for human consumption is usually evaluated in terms of the n-6/n-3 polyunsaturated fatty acid (PUFA) ratio (with optimal values ≤4). Moreover, with respect to animal production, the standard feed is unbalanced in terms of n-6/n-3 polyunsaturated fatty acids (PUFAs) with a lower proportion of the latter. Such discrepancy negatively affects the health status of animals, the productive and reproductive performances, and the general quality of their products. Hence, n-3 PUFA intake should be promoted. The increase in n-3 PUFA proportions in animal products would also be in accordance with the human dietary recommendations that often focus on the need of increasing the intake of long-chain n-3 PUFAs. In this regard, two main strategies could be implemented, namely to furnish precursor (α-linolenic acid) or long-chain derivatives (eicosapentaenoic and docosahexaenoic acids). In the present review, the effects of different n-3 PUFA sources on biological activity, physiological/reproductive endpoints, and health implications are compared focusing on the most recent results obtained in the rabbit.

**Abstract:**

This review compares the effects of different n-3 polyunsaturated fatty acid (PUFA) sources on biological activity, physiological/reproductive endpoints, and health implications with a special emphasis on a rabbit case study. Linoleic acid (LA) and α-linolenic acid (ALA) are members of two classes of PUFAs, namely the n-6 and n-3 series, which are required for normal human health. Both are considered precursors of a cascade of molecules (eicosanoids), which take part in many biological processes (inflammation, vasoconstriction/vasodilation, thromboregulation, etc.). However, their biological functions are opposite and are mainly related to the form (precursor or long-chain products) in which they were administered and to the enzyme–substrate preference. ALA is widely present in common vegetable oils and foods, marine algae, and natural herbs, whereas its long-chain PUFA derivatives are available mainly in fish and animal product origins. Recent studies have shown that the accumulation of n-3 PUFAs seems mostly to be tissue-dependent and acts in a tissue-selective manner. Furthermore, dietary n-3 PUFAs widely affect the lipid oxidation susceptibility of all tissues. In conclusion, sustainable sources of n-3 PUFAs are limited and exert a different effect about (1) the form in which they are administered, precursor or derivatives; (2) their antioxidant protections; and (3) the purpose to be achieved (health improvement, physiological and reproductive traits, metabolic pathways, etc.).

## 1. Introduction 

Polyunsaturated fatty acids (PUFAs) of the n-3 series are bioactive compounds, that exert many benefits on human health. Dietary n-3 PUFAs positively affect several physiological processes modulating health status and the onset of chronic disease, such as the regulation of plasma lipid levels [1,2], cardiovascular [3,4] and immune function [5], glucose metabolism [6], neuronal development, and visual activity [7]. Ingestion of n-3 PUFAs leads to an n-3 increase in different body tissues with effects on membrane composition and function, eicosanoid synthesis, and signaling as well as the regulation of gene expression [7,8,9,10]. n-3 PUFAs are available in some vegetable and animal sources with different chemical form and metabolic activity.

The present review aims to compare the effects of different n-3 PUFA sources on biological activity, physiological/reproductive endpoints, and health implications with a special emphasis on a rabbit case study. Rabbit, as a non-rodent model, is the smallest laboratory animal that has been well characterized, and it could be used to monitor some endpoints with relevance to humans [11,12,13].

### 1.1. Synthesis and Major Metabolic Pathways of n-3 and n-6 PUFAs

In mammals, the absence of enzymes to introduce double bonds at carbon atoms beyond C-9 in the fatty acid chain determines linoleic acid (LA, C18:2n-6) and α-linolenic acid (ALA, C18:3n-3) as essential fatty acids (EFAs) because they are not able to synthesize them. Thus, they must be included in the diet. Conversely, many animal species are able to metabolize these EFAs into long-chain (LC) derivatives (≥20 carbon atoms), namely n-3 and n-6 LC-PUFAs, which are required for normal human and animal health [14]. The number of carbon atoms from the ω end of a derived unsaturated fatty acid to the nearest double bond identifies its precursor. LA is the predominant plant-derived n-6 PUFA and is the precursor of arachidonic acid (ARA, C20:4n-6). On the other hand, ALA is the main vegetal n-3 PUFA and is the precursor of eicosapentaenoic (EPA, C20:5n-3), docosapentaenoic (DPA, C22:5n-3), and docosahexaenoic (DHA, C22:6n-3), which are the main constituents of the membrane phospholipids in nervous and reproductive tissues and gametes [7].

**Figure 1:**
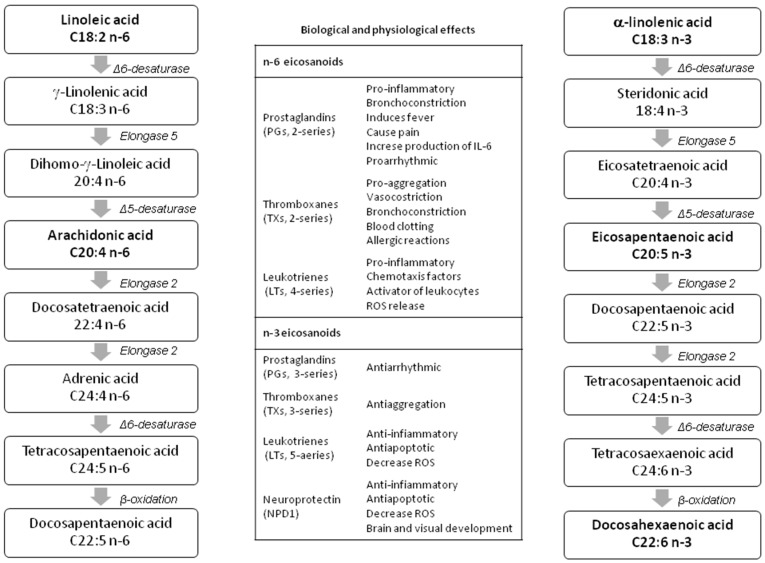
Long-chain polyunsaturated fatty acid (LC-PUFA) n-3 and n-6 biosynthetic pathways and physiological effects of their eicosanoid derivatives (modified by Patterson et al. [21]).

The same elongation (to add two-carbon units sequentially to the carboxyl end) and desaturation (to remove two hydrogen atoms from a fatty acid, creating a carbon/carbon double bond) pathways may convert LA and ALA into their long-chain metabolites [15] (see Figure 1). However, there are many differences in the PUFA metabolic pathways depending on the species—all fishes showed Δ6-desaturase activity, required for the initial desaturation of LA and ALA, whereas Δ5-desaturase, necessary to desaturate 20:4n−3 to EPA, is present only in the diadromous or freshwater species [16]. In terrestrial animals, the main monogastric species (pig, poultry, rabbit) show a certain conversion of ALA to EPA, whereas DHA synthesis is generally much lower.

Recent papers [17,18] showed that within the same species, there is a different preference for PUFA substrates. Autochthonous poultry and rabbit breeds, unselected for productive performance, desaturated more efficiently the n-3 than n-6 PUFAs [17,18]. One possible explanation of such a difference is that LA desaturation pathways are simpler than the alternative ALA route (see Figure 1); thus, it requires less metabolic energy [19,20]. 

Furthermore, it should be taken into account that the n-3 and n-6 PUFA derivatives are metabolically and functionally distinct, and have opposite physiological functions (see Figure 1), that is, n-6 PUFA derivatives have prothrombotic and proaggregatory properties, which increase blood viscosity, vasospasm, and vasoconstriction and produce decreases in bleeding time. On the contrary, the n-3 metabolites have anti-inflammatory, anti-proliferative and anti-atherosclerotic activity [22]. Consequently, it was recommended that the balance of n-6:n-3 PUFA ratio should be nearly 3:1 to 1:1 due to its importance for health and normal brain and vision tissue development, and there is a potential to enrich human and animal diets with n-3 PUFAs.

### 1.2. n-3 PUFA Sources

As it has been previously stated, ALA is widely present in common vegetable oils and foods, such as rapeseed and canola oils used in many manufactured foods, green leafy vegetables, and nuts [23]. Furthermore, many studies have investigated its presence in wild plants like marine algae [24] and spontaneous herbs (*Portulaca oleracea* L., [25]). However, the most concentrated vegetable source of ALA is linseed (*Linum usitatissimum* L.), which is largely used for industrial, food, feed, and fiber purposes. Almost every part of the linseed is utilized (seed, sprouts, oil, etc.), either directly or after processing.

Although the physiological benefits of linseed are attributed primarily to the content of ALA (about 23% in whole seed and 58% in the oil), the functional properties of linseed are not the same as pure ALA because it contains other fatty acids (FAs) (5% palmitic acid, 35% stearic acids, 17% oleic acid, and 15% linoleic acid). In addition, it is one of the richest sources of other phytochemicals, high-quality protein, and soluble fiber and phenolic compounds [26]. In particular, linseed contains a considerable number of phytoestrogens, mainly lignans and isoflavones [27], known to affect hormone and enzyme metabolism, protein synthesis, growth factors, malignant cell proliferation, and angiogenesis [28,29]. Due to these phytochemicals, linseed is receiving growing consideration to show a possible beneficial effect in reducing the risk of degenerative disease [26].

Nonetheless, most of the scientific reports show that the expected benefits of dietary n-3 PUFAs differ by animal species and are mainly associated with dietary administration of LC derivatives (EPA, DPA, and DHA), from ALA. 

Currently, fish and marine products are the main supply of n-3 LC-PUFAs [30]. The most common fish source for human consumption comes from the *Scombridae*, *Clupeidae*, and *Salmonidae* families with a high percentage of EPA and DHA. However, fish are becoming progressively scarcer and less sustainable due to the high pressures on natural fish stocks, and fish farming is unlikely to be a sustainable source because the feed used contains large amounts of wild fish [31]. 

Accordingly, new sources of n-3 LC-PUFAs are being investigated, and recently, other sources of n-3 PUFAs have been considered. For example, *Schizochytrium limacinum* is a marine microalga that can produce DHA or EPA [32]. Conchillo et al. [33] compared both microalgae oil and fish oil composition and found that DHA and EPA were the major fatty acids in them, respectively. As a result, dried *Schizochytrium* sp. has been used as a DHA-rich ingredient in the diet of broiler chicken and laying hen at levels up to 2.8% and 4.3%, respectively, with safe results [34]. 

Other marine microorganisms, which biosynthesize DHA, have been successfully cultivated, but this approach is costly and technically demanding [35]. Moreover, the factors related to the growth conditions (composition of the medium, aeration, light intensity, and temperature) affect microalgae PUFA content [35,36]. Guo et al. [37] isolated 23 yeast strains and only 9 of them integrated exogenous EPA and DHA into their cellular lipids. 

Advances in biotechnology have resulted in plants that have been genetically modified to create new compounds. In some transgenic plants, the FA metabolism has been modified to produce economical oils for food and non-food uses [38,39]. 

Furthermore, a proliferation of literature has focused on n-3 PUFA sources as animal feed to enrich animal products and improve the n-3 LC-PUFA content of food [40,41,42]. Hence, if one considers what has been previously reported, the incorporation of vegetable (mainly linseed) or fish oil in animal nutrition remains the main strategy used to improve the nutritional profile of animal products.

Focusing on rabbit production, that meat offers excellent nutritive and dietetic properties. Despite that fact, the n-6/n-3 PUFA ratio in commercial diets is frequently unbalanced with a lower proportion of the latter, and that negatively affects the PUFA ratio of the products, resulting in an over-value with respect to the optimal values recommended for human consumption [43,44,45]. Usually, the most common sources of fat in feed formulation of fattening rabbits are tallow, lard, deodorized oleins, and sunflower oil [46], which are low in n-3 PUFAs. Nonetheless, the manipulation of the rabbits’ diet has proved to be very effective in producing PUFA-enriched meat, being relatively easy to achieve optimal values [44,45,47]. 

### 1.3. Metabolism of n-3 PUFAs

**Figure 2:**
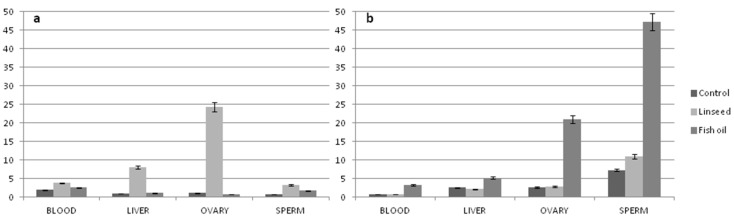
Fatty acids deposition in different tissues of rabbits fed different n-3 PUFA dietary sources (linseed or fish oil). (**a**) α-linolenic acid (ALA) distribution (% of total fatty acids) and (**b**) n-3 LC-PUFA distribution (% of total fatty acids).

ALA is converted into long-chain PUFAs by desaturation and elongation enzyme systems, as is shown in Figure 1. However, although the main metabolic site of PUFAs in mammals is the liver, recent studies have shown that the accumulation of n-3 PUFAs largely seems to be tissue-dependent and acts in a tissue-selective manner [48,49]. For instance, most of the studies show that rats fed ALA-enriched diets (using linseed, canola, and perilla oils) increased to different extents the ALA, EPA, DPA, and DHA in plasma, liver, heart, and brain [50,51]. In contrast, hearts from cardiomyopathic hamsters and hypercholesterolemic rabbits fed a diet high in ALA [52] only increased ALA and EPA [53]. In this regard, some authors have suggested that the elongase-2 activity, as well as the Δ6-desaturase, could be considered a limiting step on DHA synthesis [54]. Furthermore, recent data have demonstrated that other tissues (i.e., ovary and sperm, see Figure 2) can synthesize n-3 LC-PUFAs from a precursor in order to respond to specific needs [49]. For example, Rebollar et al., [55] found a greater deposition of EPA and DHA in the periovarian adipose tissue than in the interscapular fat of female pregnant rabbit does after a long-term dietary supplementation with fish oil. This deposition could favor the PUFA accessibility to their ovarian structures as corpora lutea, whose activity, measured by the progesterone production, was increased during the embryo preimplantation period (days 5 and 7 post-insemination) compared to females receiving no supplementation.

Furthermore, it has been described that that FADS2 gene is strongly expressed in the ovary tissue, and it is downregulated when a higher amount of n-3 LC-PUFAs is administered (i.e., fish oil-enriched diet; [49]), confirming an inhibitory effect of EPA and DHA on ALA conversion [56]. 

## 2. Different Effects of n-3 PUFAs 

### 2.1. Effect of n-3 PUFAs on Health and Cardiovascular Disease

As previously reported, n-3 PUFAs affect many physiological processes influencing normal health and chronic disease related to plasma lipid levels [2], depression [57], immune function [5], insulin action [58], neuronal development, and visual function [7]. However, the prevention of cardiovascular diseases reducing plasma triglycerides and cholesterol is probably one of the most relevant effects of n-3 [3,4].

The mechanisms whereby n-3 LC-PUFAs reduce plasma triglycerides rely on the actions of these FAs in the liver, which show (1) a lower hepatic lipogenesis and triglycerides formation; (2) a greater FA beta-oxidation; and (3) a reduction in the formation and degree of triglyceride enrichment of very low-density lipoprotein cholesterol (VLDL-c) particles. These molecular mechanisms involve modulation of several nuclear cell receptors and proteins, including peroxisome proliferator-activated receptors (PPARs), sterol regulatory element-binding proteins (SREBPs), nuclear liver X receptor α, and retinoid X receptor α [59]. The net result is less entry of VLDL-c into the circulation from the liver and enhanced clearance of circulating triglycerides.

The triglyceride-lowering properties of n-3 PUFAs are confirmed in several studies [60,61,62]. The decrease in lipid synthesis and catabolism has been suggested as the explanation of the hypotriacylglycerolaemic effect of n-3 PUFAs and the lower VLDL-c secretion [63].

Nevertheless, there is some controversy in the different reports. The expression of lipogenic genes in mice fed DHA [64] and EPA [65] was limited or had no effect in rats [66]. As it has been proven in previous works carried out in hamsters, fasting and postprandial plasma high-density lipoprotein cholesterol (HDL-c) are decreased by EPA and DHA [63]. In this regard, the decrease of total cholesterol reported in other studies in hamsters [67] and mice [68] has been related to a decrease in HDL-c and linked with overexpression of a hepatocyte structural cell membrane receptor class B, type 1 (SR-B1) [68,69]. This protein helps the uptake of cholesteryl esters from HDL-c. As a result, n-3 PUFAs could stimulate the cholesterol ester circulation to the liver, where it can either be removed in the bile or be used to create new steroid hormones [70]. 

These results have been recently corroborated in lactating rabbit females [71], which, after a long-term dietary supplementation of EPA and DHA, exhibited lower plasma levels of HDL-c and total cholesterol than those receiving no supplementation, promoting a healthier lipid plasma profile for them. 

On the other hand, n-3 PUFA regulation is not only done by hepatocytes, but also by adipose tissue, which modulates FA oxidation and/or the secretion of several hormones such as leptin. It is one essential hormone secreted by adipose tissue, which could be affected by changes in the FA profile of the diet. The role of this hormone in hypothalamic-mediated appetite suppression in response to caloric intake is not the only activity. Fatty acids might regulate transcription of leptin and several adipocyte-specific genes by changing the regulation pattern of the nuclear receptor PPARγ [72]. A study on rats fed fish oil found that the epididymal leptin mRNA levels decreased by increasing activation of PPARγ [73]. In contrast, some research studies on pregnant and lactating rabbit females have confirmed a higher leptimenia after the inclusion of EPA and DHA in their diet compared to a control group [71].

Another adipose tissue that is involved in thermogenesis, which protects the body from a cold environment by dissipating the chemical energy of lipid and glucose, is brown adipose tissue (BAT). In rabbits, BAT is principally located in the interscapular and perirenal fat [74], and larger amounts of BAT are present in newborns and decrease with age [75,76]. At birth, the majority of mammals, but especially the precocial species [77], have to be adapted to the low temperature of the extra-uterine environment. Thus, the thermogenesis in BAT is necessary for the effective adaptation to this environment. Moreover, altricial newborns, such as mice, rats, and rabbits, are born after a short gestation period and without a hair cover, depending on the lipids from BAT to increase their FA β-oxidation and heat production [78]. In this regard, researchers have described an important hyperlipidemia in the newborns from rabbit does that were fed a diet enriched in EPA and DHA during pregnancy. In addition, a greater oxidation of these n-3 PUFAs was also observed in the kits, which could indicate an improvement of their survival during their first hours of life thanks to the heat production [71]. 

### 2.2. Effect of n-3 PUFAs on the Digestive System

Dietary FAs could affect the intestinal microbiota of animals. In rabbits, Marounek et al. [79] observed that dietary FAs affected cecal fermentation and decreased the development of pathogenic strains. It seems that there is a relationship between the enrichment of diet with medium-chain FAs (caprylic and capric) and the alteration of intestinal microbiota [80]. In a study carried out supplementing the diet of growing rabbits with EPA and DHA [44], most of the parameters regarding cecal fermentation (pH, dry matter) were not affected by supplementation. However, although the reason is unclear, the concentration of total cecal volatile fatty acids was greater in those rabbits having a diet enriched with EPA and DHA. In a later study, feeding early-weaned rabbits (25 days old) with the same diets, these differences were not observed [81]. 

At the ileal level, a reduction in their intestinal villous/crypt height ratio could indicate damage of the gut barrier function. This process can be induced by the change of diets carried out during the weaning of animals. In studies performed during the growing period [82], young rabbits supplemented with EPA and DHA from fish oil did not show differences in both their values of crypt depth and villi length, which had normal dimensions [83,84]. 

Accordingly, some human studies have showed that the anti-inflammatory benefits of n-3 PUFAs on gut microbiome may be attributed to DHA metabolites, in particular, those resulting from endogenous lipoxygenase-catalyzed hydroxylation of DHA, which in turn produces resolvins and protectin D1 through acetylation of the cyclooxygenase-2 enzyme [84,85]. Numerous reports describe the protective effects of EPA- and DHA-derived mediators in experimental models of inflammatory bowel diseases [86]. In young rabbits naturally affected by epizootic rabbit enteropathy and fed an enriched n-3 diet using linseed, Maertens et al. [87] observed a reduced mortality with respect to control animals.

It should be underlined, in general terms, that several factors affect the composition of the microbiota, for example, diet consumed, stress situation, antibiotic therapy, or environmental exposure to microorganisms [88]. In this regard, Rodríguez et al. [44], using EPA and DHA in the diets of growing rabbits, did not observe any improvements in their gastrointestinal health, probably due to the optimal ambient and sanitary conditions of the experimental farm where the study was carried out. However, in a study with similar ambient conditions [81] and using the same enriched diet, a stressful situation promoted by an early weaning adversely affected the animals having no supplementation, resulting in a higher morbidity (lethargy/weakness, crouched posture, rough coat) with respect to EPA- and DHA-supplemented growing rabbits. 

### 2.3. Effect of n-3 PUFAs on Reproductive Performance 

As already stated, n-3 PUFAs affect several aspects of reproductive activity, influencing both male (sperm quality, hormone profile) and female (ovarian functions, hormone response, oviduct and uterus environment, tissue FA profile) traits (see Table 1).

Sperm cells of rabbit fed fish oil show a higher n-3 LC-PUFA content when compared to blood, liver, and ovary (see Figure 2; [49]). Such an accumulation of n-3 PUFA in rabbit sperm is probably due to the demand of the sperm, whose membrane consists mainly of PUFAs (more than 60% of sperm membrane FAs are LC-PUFA, [89,90]).

In agreement, Mourvaki et al. [90] found that the integration of 5% linseed into the rabbit diet improves sperm quality by modifying the sperm lipid composition, which means a reduction by half of LA and DPA (22:5n-6), and a concomitant increase of ALA (+ 65%) and DHA (+ 83%). Furthermore, the author also found a 70% reduction in sperm cholesterol. The relevant increase in DHA, mainly in the sperm tail, as well as the similar decrease in cholesterol, can influence sperm speed and the fluidity of their membranes improving the quality traits of semen. This positive effect is probably due to the lipid bilayer of the sperm membrane consisting mainly of phospholipids with considerable amounts of LC-PUFAs [89,91]. The phospholipid-bound PUFAs, along with cholesterol, are responsible for changes in sperm membrane fluidity and thus may regulate acrosome responsiveness and oocyte-sperm fusion [92]. Furthermore, Castellini et al. [93] demonstrated that dietary linseed supplementation on rabbit bucks improves the motility rate and speed track of sperm (curvilinear velocity, VCL), and increases the blood testosterone concentration resulting in higher fertility. However, the positive effect on male rabbits is only found with concomitant intake of antioxidant compounds (vitamin E and/or vitamin C). In agreement, Gliozzi et al. [94] demonstrated that the semen quality of rabbits fed a fish oil-enriched diet was strongly different if vitamin E was also provided, that is, rabbit fed fish oil diets (both with and without vitamin E) showed about 5-fold higher values of DHA in semen phospholipids. Nevertheless, many other studies on male reproduction (in rabbit [90,95,96]; in stallion semen [97]; in ovine [98]) highlighted the importance of simultaneous antioxidant protection in addition to dietary n-3 PUFAs (see Table 1). 

Regarding the female diets, it is well documented that the addition of n-3 PUFAs affects the circulating concentrations of PUFA metabolites and hormones, influencing the growth factors on the follicular fluid, as well as in the oviduct and in the uterus [99,100]. In this regard, studying the n-3 PUFA profile of female reproductive tissues, the ovary of rabbit fed fish oil or linseed dietary supplementation shows a higher proportion of n-3 PUFAs with respect to the blood and liver (see Figure 2). Furthermore, the highest accumulation of n-3 derivatives (EPA, DPA, and DHA) has been registered with supplementation of fish oil and not with linseed (see Figure 2b). Such a result was justified by the lower expression of FADS2 gene, which encoded for the Δ6-desaturase enzyme [49], and suggests that some tissues, although maintaining a certain metabolic activity, do not reach suitable levels in order to respond to specific needs.

Concerning ovarian function and follicular environment, it has been described that oocytes of many animal species use the high levels of FAs that compose them as an energy source during their process of maturation and embryo development before implantation. Kim et al. [50] showed in a study carried out in cows that the composition of cumulus cells, granulosa cells, and oocytes, in terms of FAs, was altered by dietary PUFAs. This fact may be relevant for oocyte quality, maturation, and subsequent competence [101]. Recent results [102] in artificially inseminated rabbit females confirmed that a long-term dietary enrichment with EPA and DHA improved the quality of their blastocysts in terms of the apoptosis rate.

On the other hand, the position of the double bond in the carbon chain determines the ability of PUFAs to act as precursors of other essential compounds (e.g., hormones, cytokines). In the pre- and post-implantation period of rabbit females (days 5–7 and 7–14 post-artificial insemination (AI)), the supplementation with DHA and EPA results in an increase in plasma progesterone [56,102]). The higher the progesterone concentration, the better implantation and placentation process take place, and consequently, a higher survival post-implantation of fetuses occurs [103]. Some studies [104,105] explain this improvement by a reduction of the 2-series prostaglandin (PG) derivatives production from n-6 PUFAs (see Figure 1). Precisely, a low uterine secretion of PGF_2_α during early embryonic development could avoid the start of luteolysis and thereby promote the establishment of pregnancy [106], giving the conceptus longer to grow before the possible luteal regression [107]. 

In primiparous rabbit does, when the second artificial insemination was performed, the fertility rate decreased drastically due mainly to a poor body condition [108]. As previously mentioned, the inclusion of EPA and DHA in the diet of primiparous lactating rabbit does increases plasma leptin and estradiol concentrations during lactation [71]. These findings could indicate an adequate body condition and sexual receptivity in these rabbit females, which in turn could improve their fertility rate in following inseminations. The current hypothesis was confirmed, and when primiparous does fed over the long term with DHA- and EPA-supplemented diets were inseminated, a greater fertility rate was obtained compared to primiparous does fed a control diet [55].

Moreover, PUFAs can wield a direct influence during the different phases of gestation, facilitating the placenta blood flow to the fetus and, consequently, improving fetal development and growth. This is possible since the ratio between prostacyclin and thromboxane is increased by n-3 PUFAs and, as a result, this promotes vasodilatation and the reduction of blood viscosity [109]. In this regard, although no effect was observed in the percentage of viable fetuses, Rodríguez et al. [110] found greater fetoplacental development in terms of fetus size and placental efficiency when EPA and DHA were used in rabbit does’ diets, and consequently larger and heavier newborns were found [71,102,103]. Furthermore, the positive effect of PUFA supplementation on the survival ability of rabbit kits has been documented. Several evidences from animal models and humans suggest that dietary n-3 LC-PUFAs during gestation promote early brain development and regulate behavioral and neurochemical aspects related to stress responses, aggression, growth, and cognitive functions [111,112].

The second way by which newborn mammals can benefit from n-3 PUFA supplementation is when litters consume colostrum and milk containing elevated concentrations of EPA and DHA. According to Lin et al. [113], rabbit milk composition obtained with standard diets may be too low in DHA to meet the need of a growing rabbit. In this regard, Rodríguez et al. [102] demonstrated that the amount of n-3 LC-PUFAs in the milk increases when the diet of lactating does is enriched with EPA and DHA.

### 2.4. Effect of n-3 PUFAs on Oxidative Stress 

The imbalance between the levels of reactive oxygen species (ROS) and cellular antioxidants (both intra- and extra-cellular) is known in general terms as oxidative stress situation. In this situation, the amount of ROS in the organism is excessive [114].

Low and moderate quantities of ROS are beneficial for some physiological processes, including pathogen elimination, tissue repair, and wound healing. Nonetheless, under oxidative stress situation, in which the amount of ROS is too high, this also provokes the oxidative damage of DNA, RNA, lipids (including PUFAs), and proteins. As a result of these damages, a specific toxicity can occur of both organs and pathways related to several biological processes, such as alterations of membrane permeability, the encouragement of apoptosis, or the reduction of the antioxidant defense of the body [116,117].

One of the main problems on livestock production is generated by stress situations. For example, the moment of weaning, some environmental factors, as well as some illnesses and infections can generate pro-oxidant compounds that induce oxidative stress [118]. The age of animals is also important in their oxidative status. In this regard, newborns are more prone to develop oxidative stress than adults, due to the change of breathing [119]. The transition from the intra-uterine to the extra-uterine environment implies a sharp increase of oxygen concentration around the animal and, consequently, a precipitated pulmonary adaptation to the post-natal condition is needed [119]. These first breaths of neonates generate a high concentration of ROS [120]. 

Rodríguez et al. [71], who found higher oxidative stress in rabbit neonates than in their mothers, have recently confirmed these statements. Additionally, in ewes, Rizzo et al. [121] found a significant increase in serum ROS concentrations between 36 h before and 24 h after lambing, and other reports confirmed an increased risk of oxidative stress in newborn calves [120,122]. 

Following this line of investigation, Cavaliere et al. [123] evaluated in a rat model the properties of milk produced by dairy cows fed a diet characterized by a high forage:concentrate ratio and a low n-6:n-3 ratio and high content of conjugated linoleic acid. These results positively affected lipid metabolism, leptin:adiponectin ratio, inflammation, mitochondrial function, and oxidative stress. Subsequently, Trinchese et al. [124] demonstrated that the supplementation of rats’ diet with this high content of conjugated linoleic acid results in a reduced lipid content and inflammation levels in the skeletal muscle of these animals, and an improved mitochondrial lipid oxidation and redox status through modulation of AMP-activated protein kinase activity.

Furthermore, oxidation is an accelerated chain reaction. The susceptibility of FAs to oxidation is directly dependent on the degree of unsaturation; subsequently, supplementations with highly unsaturated n-3 PUFAs considerably increase oxidative damage [125]. Recent studies in rabbit confirmed oxidative stress after the inclusion of dietary fish oil in both females does and newborns, where it was even more pronounced [71]. 

The same lipid oxidation susceptibility could affect the male reproductive traits and, in particular, the semen quality of animals feeding on n-3 PUFAs, considering that dietary source influences the PUFA profile of sperm (see Section 2.3). In agreement, recent studies (reviewed by [126]) demonstrated that lipid membrane peroxidation generates a series of molecules, namely isoprostanes, which are retained “markers of oxidation” in many male infertility-related pathologies [127] or neurodegenerative disease (i.e., Rett syndrome or Alzheimer’s disease [128]). Isoprostanes are prostaglandin-like products, which are formed via nonenzymatic, free radical-mediated peroxidation of polyunsaturated fatty acids, for example, the oxidation of arachidonic acid (n-6 LC-PUFA) produces F2-isoprostanes (F2-IsoPs), whereas the oxidation of EPA and DHA (n-3 LC-PUFAs) produces F3-isoprostanes (F3-IsoPs) and F4-neuroprostanes (F4-NeuroPs), respectively [129]. Their distribution depends on PUFA precursor localization and it changes with the fatty acid composition of tissues. It was demonstrated that a diet rich in n-3 PUFAs increases the concentration of F4-NeuroPs in the blood and seminal plasma, while reducing that of F2-IsoPs (unpublished data).

Losano et al. [130] in bull sperm demonstrated that PUFA supplementation negatively affects the semen quality when it was provided without additional vitamin E because the sperm cell membrane can easily oxidize. Hence, many authors agree that a PUFA-enriched diet, regardless of the reason for which it is administered, requires antioxidant protection (i.e., vitamin E, vitamin C, selenium; [41,114,129]) in order to avoid the onset of oxidation.

Studies on linseed supplementation on rabbit meat confirmed what was previously reported. Petracci et al. [129], using dietary linseed, found three times higher α-linolenic content in rabbit meat; however, the higher level of PUFAs also induced higher susceptibility to lipid oxidation. In agreement, Dal Bosco et al. [41] observed a positive effect of dietary α-tocopheryl acetate (up to 289 mg/kg) on the lipid oxidation of meat obtained from rabbits fed a diet containing 8% linseed. Then, further antioxidant protection is strongly recommended when diets enriched in PUFA are provided to the animals.

## 3. Conclusions

n-3 PUFAs are bioactive compounds essential for the health of all living organisms. However, sustainable sources of these compounds are limited and exert a different effect in relation to the form in which the n-3 PUFAs are provided (precursor (ALA) or derivatives (EPA, DPA, DHA)). In summary, considering the rabbit as a case study, positive effects were described in lipid metabolism and steroid hormone secretion in females, as well as favorable repercussions in reproductive tissues and gametes of both males and females, which are a prerequisite to achieving good fertility rates and offspring survival.

## Figures and Tables

**Table 1:** Effect of n-3 PUFA dietary supplementation with (+ vit E) or without (– vit E) an appropriate antioxidant protection (200 mg/kg vitamin E), on rabbit reproductive parameters.

Item	n-3 PUFA Precursor (ALA)	n-3 PUFA Products (EPA/DHA)
Male		
Performance	↑ Blood testosterone concentration [96], ± vit E ↑ Sperm motility rate and track speed (VCL) [92], + vit E ↓Sperm motility rate and track speed (VCL) [92], − vit E	↑ Acrosome reaction [114], − vit E ↓ Live and motile (ALH) cells [114], − vit E ↓Sperm motility rate and track speed (VCL) [89], − vit E
Tissues	↑ Sperm membrane fluidity [92], + vit E ↑ Sperm EPA and DHA concentration [92] + vit E ↓ Sperm membrane cholesterol [92,93], + vit E	↑ Production of ROMs and TBARS [114], ± vit E ↑ Sperm EPA and DHA concentration [94,95] ± vit E ↓ Sperm ARA concentration [94,95] ± vit E ↓ Semen antioxidant capacity [114], ± vit E
Female		
Performance	↑ Fetuses’ survival [72,115], ± vit E	↑ Fertility rate [102] ± vit E ↑ Progesterone in the pre- and post-implantation periods [102,103] ± vit E ↑ Fetuses’ survival and growth [103] ± vit E ↓ Luteolysis [103] ± vit E
Tissues	= Ovarian PUFA composition [48], ± vit E	↑ LC-PUFA deposition in ovarian [49], ± vit E ↑ LC-PUFA deposition in periovarian adipose tissue [103] ± vit E ↑ Placental development [111] ± vit E ↓ Apoptotic rate of blastocysts [102] ± vit E

ALH: amplitude of lateral head displacement; ROMs: reactive oxygen metabolites; TBARS: thiobarbituric acid reactive substances.
